# High-throughput sequencing of circRNAs reveals novel insights into mechanisms of nigericin in pancreatic cancer

**DOI:** 10.1186/s12864-019-6032-3

**Published:** 2019-09-18

**Authors:** Zhihua Xu, Jiaqing Shen, Shangbo Hua, Daiwei Wan, Qian Chen, Ye Han, Rui Ren, Fei Liu, Zhiyong Du, Xiaobo Guo, Jianming Shi, Qiaoming Zhi

**Affiliations:** 1 Department of General Surgery, The First Affiliated Hospital of Soochow University, Suzhou, 215006 China; 2 Department of Gastroenterology, The First Affiliated Hospital of Soochow University, Suzhou, 215006 China; 3 Department of General Surgery, Kunshan Hospital of Traditional Chinese Medicine, Kunshan Affiliated Hospital of Nanjing University of Chinese Medicine, Kunshan, 215300 China; 4 Department of Oncology, Suzhou Municipal Hospital, Nanjing Medical University Affiliated Suzhou Hospital, Suzhou, 215002 China; 5 Department of General Surgery, The Second Affiliated Hospital of Soochow University, Suzhou, 215000 China; 6 Department of Hepatobiliary and Pancreatic Surgery, Shenzhen People’s Hospital, Shenzhen, 518020 China; 7 Department of Gastrointestinal Surgery, Provincial Hospital Affiliated to Shandong University, Jinan, 250021 China

**Keywords:** Nigericin, High-throughput sequencing, CircRNAs, Mechanism, Pancreatic cancer

## Abstract

**Background:**

Our previous study had proved that nigericin could reduce colorectal cancer cell proliferation in dose- and time-dependent manners by targeting Wnt/β-catenin signaling. To better elucidate its potential anti-cancer mechanism, two pancreatic cancer (PC) cell lines were exposed to increasing concentrations of nigericin for different time periods, and the high-throughput sequencing was performed to explore the circRNA expression profiles after nigericin exposure on pancreatic cancer (PC) cells.

**Results:**

In this study, a total of 183 common differentially expressed circRNAs were identified, and the reliability and validity of the sequencing data were verified by the PCR analysis. According to the parental genes of circRNAs, the GO analysis was performed to predict the most enriched terms in the biological process, cellular components and molecular functions. The KEGG analysis and pathway-pathway network exhibited the potential signal pathways and their regulatory relationships. Meanwhile, a potential competing endogenous RNA (ceRNA) mechanism through a circRNA-miRNA-mRNA network was applied to annotate potential functions of these common differentially expressed circRNAs, and these predicted miRNAs or mRNAs might be involved in nigericin damage.

**Conclusions:**

By the bioinformatics method, our data will facilitate the understanding of nigericin in PC cells, and provide new insight into the molecular mechanism of nigericin toward cancer cells. This is the first report that discusses the potential functions of nigericin in cancers through the bioinformatics method. Our data will facilitate the understanding of nigericin-mediated anti-cancer mechanisms in PC.

**Electronic supplementary material:**

The online version of this article (10.1186/s12864-019-6032-3) contains supplementary material, which is available to authorized users.

## Background

Nigericin is an ionophorous antibiotic derived from Streptomyces hygroscopicus and originally identified as a K/H antiporter, by which mechanism intracellular pH level is maintained by the Na/H antiporter that excludes H from the cells in exchange for Na [1]. Nigericin is of great interest since in addition to producing a prominent hyperpolarization, and exerts more significant effect on generation of electrically induced action potential than other ionophores antiporter [2]. As a K^+^, H^+^-ionophore with antibiotic [3], antimalarial [4], anti-Toxoplasma gondii [5] and antiviral potency [6], nigericin has been also considered for a potential anti-cancer drug of malignancy, though it has not been translated into clinical trials until now. It was found to inhibit DNA synthesis of cancer cells by increasing intracellular pH and causing acidification of cytoplasm [7]. In 2009, Gupta et al. demonstrated that nigericin had structural similarity to salinomycin and exhibited selective toxicity to breast stem cells [8]. In 2012, Zhou et al. reported that nigericin could suppress colorectal cancer metastasis by partly reversing the epithelial-mesenchymal transition during cell invasion and metastasis [9]. Deng et al. showed that nigericin could selectively target cancer stem cells in nasopharyngeal carcinoma both in vitro and in vivo. Nigericin decreased invasion and migration of nasopharyngeal cancer cells and enhanced the cytotoxic effects of the traditional chemotherapy [10]. Our recent study in 2018 also proved that nigericin treatment significantly reduced tumor cell proliferation in dose- and time-dependent manners in colorectal cancer cells by targeting Wnt/β-catenin signaling [11]. However, the role of nigericin and its potential mechanism in cancers have not been fully elucidated.

Most precursor mRNAs (pre-mRNAs) experience a canonical manner of splicing, which exons are joined from 5′ to 3′ to generate linear mRNAs that can be translated into proteins subsequently. However, emerging evidence has revealed that pre-mRNAs undergo back-splicing and join a splice donor to an upstream splice acceptor, in which manner of producing circular RNAs (circRNAs). CircRNAs are a kind of non-coding transcripts that posses the form of a covalently closed continuous loop where the 3′ and 5′ RNA ends are joined together. Because of the feature of covalently closed loop, circRNAs are more stable than linear RNAs that they are not easily digested by traditional RNA exonuclease [12, 13]. CircRNAs can accomplish a remarkable multitude of biological functions in mammals, such as regulating parental gene transcription [14], serving as miRNA sponges [15, 16] or interacting with RNA-binding proteins [17, 18]. Additionally, circRNAs are also demonstrated to have protein-coding potential and expand the eukaryotic proteome [19, 20]. These studies provide the pathological molecular mechanisms and direct new strategies for disease treatment and diagnosis. For example, Zhang et al. implied that ci-ankrd52, which was a circular intronic RNA (ciRNA), could function as positive regulators of Pol II transcription and play a cis-regulatory role in the efficient transcription of its parental gene in Hela and H9 cells [21]. In 2017, a study from Weng et al. demonstrated that ciRS-7 was significantly up-regulated in colorectal cancer tissues, which could sponge miR-7 to activate EGFR and RAF1 activity [22]. Chen et al. performed experiments in vitro and in vivo and proved that circEPSTI1 could bind to miR-4753 and miR-6809 as a miRNA sponge to regulate BCL11A expression and affect triple-negative breast cancer proliferation and apoptosis [23].

Besides, circRNAs also have attracted the attention of more researchers and are found to be involved in the biogenesis and development of pancreatic cancer (PC). In 2016, a high-throughput circRNA microarray in six cancer samples and paired adjacent normal tissues was performed by Li et al. to identify dys-regulated circRNAs in pancreatic ductal adenocarcinoma patients [24]. The results of Chen et al. suggested that circRNA_100782 regulated BxPC3 cell proliferation by acting as miR-124 sponge through the IL6-STAT3 pathway [25]. A recent study from Huang et al. found that silencing hsa_circ_0000977 could suppress the progression of PC by interacting with hsa-miR-874-3p and decreasing inhibiting PLK1 expression [26]. To our knowledge, there are no data assessing the potential role of nigericin in PC. Therefore, in this study, PC cells were exposed to different concentrations (0, 0.25, 0.5, 1, 2, 5, 10, 20 and 50 μmol/L) of nigericin for different time periods (0, 8, 16 or 32 h) respectively, and the high-throughput sequencing was performed to explore the circRNA expression profiles after nigericin exposure at different time points. Subsequently, from the perspective of circRNAs through regulating parental gene transcriptions or serving as miRNA sponges, we used the bioinformatics analyses to explore the potential mechanism of nigericin in PC cells. Our new finding will facilitate the understanding of nigericin-mediated potential anti-cancer mechanisms in PC.

## Methods

### Cell culture and reagents

Human PC cell lines (PANC-1 and SW1990) were purchased from Shanghai Institute of Biochemistry and Cell Biology at the Chinese Academy of Sciences (Shanghai, China). Cells were cultured in Dulbecco’s Modified Eagle Medium (DMEM, Gibco) supplemented with 10% fetal bovine serum (FBS, Gibco) at 37 °C in a humidified incubator containing 5% CO_2_. Cells were in the logarithmic phase of growth for all experiments. Nigericin was purchased from Sigma Aldrich (USA). The stock solutions (100 mmol/L) were prepared with DMSO and stored at − 37 °C.

### MTT assay

Cell proliferation of PANC-1 and SW1990 cells with or without nigericin treatment was analyzed by 3-(4,5- Dimethyltjiazol-2-yl)-2,5-diphenltetrazolium bromide (MTT) assay. Cells were seeded in a 96-well plate at a density of 3 × 10^4^ cells per well and then incubated at 37 °C for 24 h. At 0, 8, 16 and 32 h after nigericin treatment with different concentrations (0, 0.25, 0.5, 1, 2, 5, 10, 20 and 50 μmol/L), cells were incubated with 20 μl of 5 mg/ml MTT (Sigma, Shanghai, China) for 4 h at 37 °C, followed by treated with 200 μl of dimethyl sulfoxide (DMSO) for 20 min. The absorbance of each well was measured with a Microplate Reader (Epoch, Winooski, USA) under 490 nm. The experiment was performed in triplicate and repeated three times.

### High-throughput RNA sequencing analysis

Cells were exposed to a certain concentration of nigericin (5 μmol/L) for different time periods (0, 8, 16 or 32 h), and then total RNA was extracted from cells respectively. The quantity and integrity of total RNAs were measured by the NanoDrop™ ND-2000 (Thermo Fisher Scientifc, Scotts Valley, CA, USA) and the Agilent Bioanalyzer 2100 (Agilent Technologies, Santa Clara, CA, USA) respectively. CircRNAs were quantitatively analyzed by Shanghai OE Biotech (Shanghai, China). After removal of ribosomal RNA and then constructing a library, a high-throughput RNA sequencing was performed. The clean reads were aligned to the reference genome by Bowtie2 (http://bowtie-bio.sourceforge.net/bowtie2/manual.shtml). For unmapped reads, the junctions were picked out using back-splice algorithm. Finally, circRNAs were verified with software developed by Shanghai OE Biotech which was considered as the reference sequence for further analysis. The expression levels of circRNAs were measured by RPM (“Mapped backsplicing junction reads per million mapped reads”). High-throughput sequencing data reported herein had been deposited at the NCBI website (https://www.ncbi.nlm.nih.gov/Traces/study/?acc=PRJNA543685&go=go) with the accession number PRJNA543685 (SRR9107550, SRR9107551, SRR9107552 and SRR9107553).

### Differentially expressed circRNA screen and clustering analysis

Differentially expressed circRNAs were detected by the negative binomial distribution test based on the DESeq package. These circRNAs with statistical significance were screened with p-value less than 0.05, false discovery rate (FDR) less than 0.05 and fold change (FC) more than 2.0. Linear transcripts were annotated according to the location of the chromosome where the circRNA sequence was overlapped. Comparing the circRNA with genetic elements, the circRNA distribution in the genome could be explored. Venn analysis was used to show the common characteristic elements among 3 comparision groups (0 h vs 8 h, 0 h vs 16 h, 0 h vs 32 h). The common differentially expressed circRNAs were showed in pies with different colors. The non-supervised hierarchical clustering was used in the form of heat map to display the expression patterns of the differentially expressed circRNAs.

### Quantitative real-time PCR (qRT-PCR) validation

To verify the reliability of the high-throughput RNA sequencing, the expression level of circRNAs was detected by qRT-PCR. The Primer Express software version 5.0 was used to design the specific primers for each circRNAs in Additional file 1: Table S1. Total RNA was extracted, digested using RNase R and purified, cDNA was synthesized using the Prime Script RT Master Mix (Takara, Japan). Outward-facing primers were designed to amplify the fragment across the junction from cDNA, and the fragment was sequenced by Sangon Biotech (Shanghai, China). QRT-PCR was performed using the SYBR green mix (Roche, Mannheim, Germany), and PCR-specific amplification was conducted in LightCycler 96 System (Roche). The expression of circRNAs was defined based on the threshold cycle (Ct), and relative expression levels were calculated through the 2^-ΔΔCt^ method. GAPDH served as internal standard control, and all reactions were performed in triplicate.

### The gene ontology (GO) and Kyoto encyclopedia of genes and genomes (KEGG) pathway analysis

Emerging reports have shown that there is a close association between circRNAs and their parental genes [14]. A study from Wei et al. in 2017 demonstrated that there was a strict linear relationship between circRNAs and their parental genes, which indicated that circRNAs were generated as by-products of linear mRNA [27]. Besides, various circRNA isoforms can be transcribed from the same parental gene locus, and the functions of circRNAs may be associated with those parental linear transcripts [28]. Recently, Wang et al. analyzed the parental genes of the dys-regulated circRNAs between invasion and noninvasion nonfunctioning pituitary adenomas by the GO and KEGG enrichment analysis, and found that some cell adhesion signaling pathways such as Focal adhesion, Hippo signaling pathway, PI3K-Akt signaling pathway and Adherens junction were enriched [29]. Therefore, the bioinformatics analysis of parental genes including the GO and KEGG enrichment analysis may partly explain some functions of these dys-regulated circRNAs. The GO analysis was conducted to construct meaningful annotations of genes and gene products in a wide variety of organisms through DAVID database (http://david. abcc.ncifcrf.gov). Our data provided an ontology of potential functions of the linear transcripts, and covered three domains: cellular components, biological process and molecular function. The top 10 enriched GO terms among 3 comparison groups were presented. The KEGG pathway analysis was adopted to determine the involvement of linear transcripts in different biological pathways. The top 20 enriched pathways were also described. Based on the results of enrichment analysis, the interaction between KEGG pathways was analyzed. A KEGG pathway-pathway network was conducted to explore the upstream and downstream signal pathways.

### Prediction of circRNA and miRNA interactions

Putative interactions between circRNAs and miRNAs were evaluated through miRanda (http://www.microrna.org/microrna/home.do), investigating only perfect seed matching without gap of Wooble pairing (‘strict’ parameter). A hit between any expressed miRNA (including the new predicted miRNA) and a target circRNA was considered for a miRanda score of 140 or higher, corresponding to at least a perfect seed match. To establish circRNA-miRNA network, we searched for miRNA response elements (MREs) on circRNAs using the software, and selected the miRNAs according to seed match sequences.

### Competing endogenous RNA (CeRNA) network analysis

The common differentially expressed circRNAs were subjected to the analysis. Putative targets of circRNAs were predicted by the method as described above. Putative targets of miRNAs were predicted by means of Targetscan (http://www.targetscan.org/). To further investigate the functional roles of those common differentially expressed circRNAs, the ceRNA network was conducted to predict the potential circRNA-miRNA-mRNA interactions.

### Statistical analysis

A statistical analysis was performed using Student’s *t*-test to compare two variables of the sequencing data. The differences with fold change (FC) ≥2.0 and *P* < 0.05 were considered to be statistically significant. The false discovery rate (FDR) was calculated to evaluate the significance of the *P* value.

## Results

### Sensitivity of human PC cells to nigericin

**Fig. 1:**
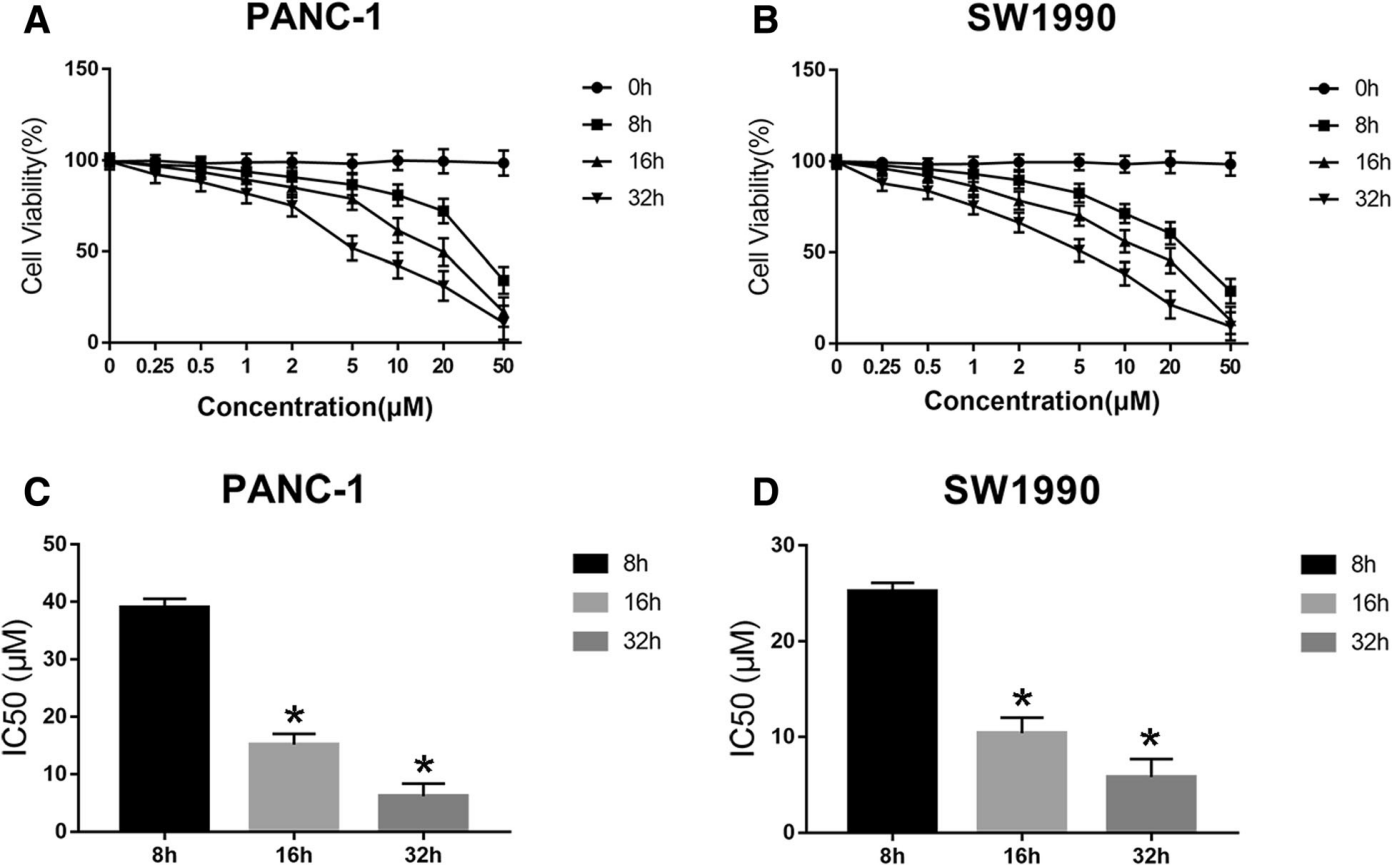
Sensitivity of human PC cells to nigericin. **a, b** PANC-1 and SW1990 cells were exposed to increasing concentrations of nigericin for different time periods (0, 8, 16 or 32 h), and nigericin treatment significantly reduced the cell viability in a concentration-dependent manner. **c, d** The IC50 values of nigericin in PANC-1 and SW1990 cells at different time points (8, 16 and 32 h) were calculated and compared. Nigericin inhibited the cell viabilities time dependently. (*P < 0.05)

As shown in Fig. 1a, b, nigericin treatment significantly decreased tumor cell viability in a concentration-dependent manner in PANC-1 and SW1990 cells. Moreover, the IC50 values of nigericin were calculated in the two cell lines at different time points (8, 16 and 32 h). The results demonstrated that nigericin also inhibited the cell viability time dependently (39.99 ± 1.53, 15.14 ± 1.90 and 6.21 ± 2.18 μmol/L in PANC-1 cells; 25.21 ± 1.18, 10.38 ± 1.62 and 5.79 ± 1.81 μmol/L in SW1990 cells at 8, 16 and 32 h (Fig. 1c, d, *P < 0.05), indicating the anti-cancer properties of nigericin in PC.

### The differentially expressed circRNA profile by high-throughput sequencing

**Fig. 2:**
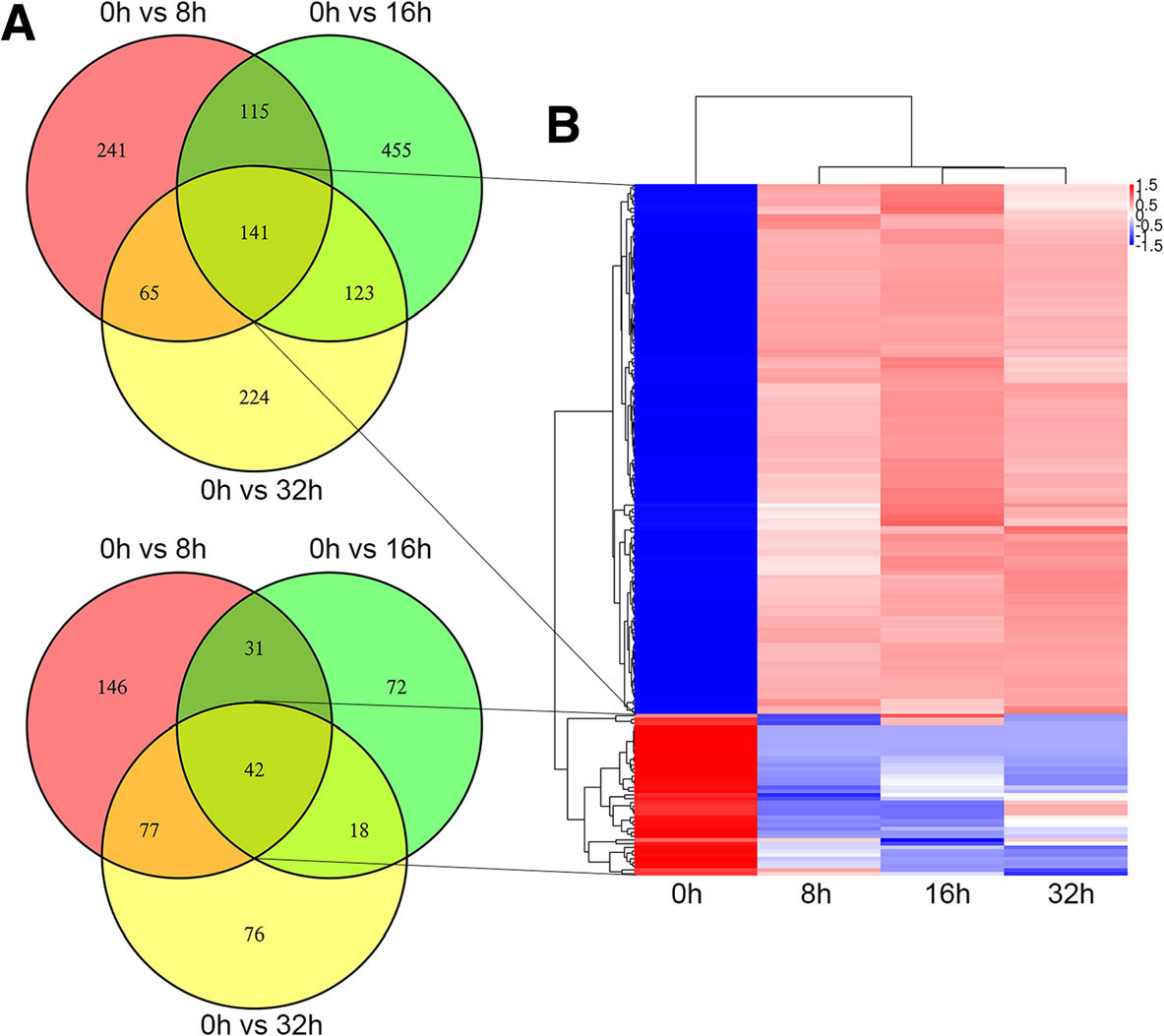
The common differentially expressed circRNAs during nigericin treatment were indentified. **a** The venn analysis among the three comparison groups showed that 183 common differentially expressed circRNAs (141 up-regulated and 42 down-regulated ones) were involved in the nigericin treatment. **b** The cluster heat map of these common differentially expressed circRNAs was presented

**Fig. 3:**
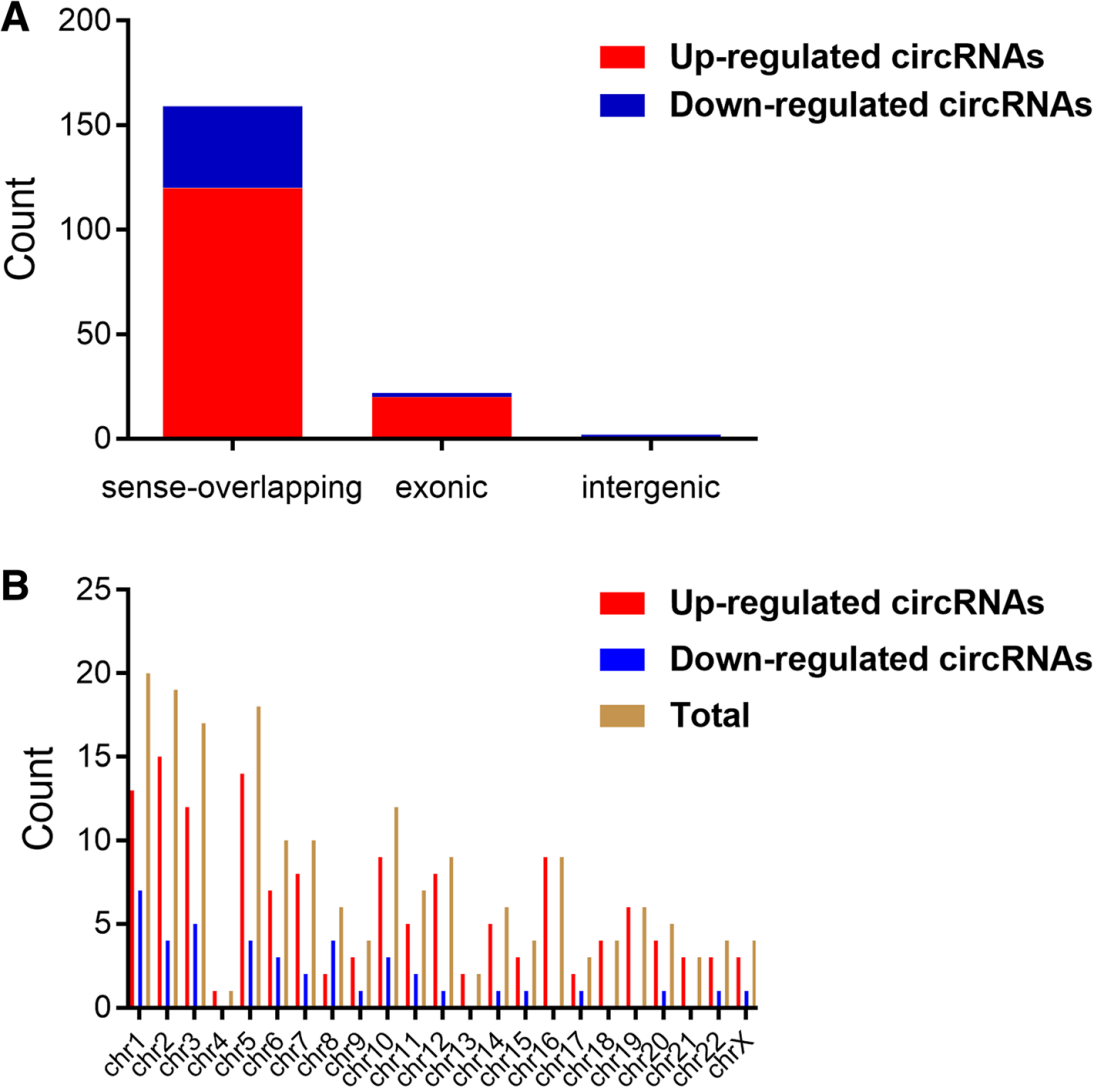
The characterizations of the common differentially expressed circRNAs. **a** Analyzing these 183 circRNAs, 159 ones were sense-overlapping, 22 ones were derived from exons, and only 2 circRNAs were intergenic. **b** The distribution of the common differentially expressed circRNAs on the human chromosomes was depicted

We used the high-throughput RNA sequencing analysis to reveal the expression profile of circRNAs in PC cells after nigericin treatment. The hierarchical clustering analysis in the form of heat map exhibited the differentially expressed circRNAs between different time periods (0, 8, 16 and 32 h) (Additional file 2: Figure S1A). Three comparison groups (0 h vs 8 h, 0 h vs 16 h and 0 h vs 32 h) were set, and the scatter plots were used to investigate the circRNA expression profiles in each comparison group (Additional file 2: Figure S1B). 562 circRNAs were up-regulated and 296 ones were down-regulated after 8 h-nigericin treatment. In the comparison group of 0 h vs 16 h, 834 circRNAs were up-regulated and 163 ones were down-regulated. Similarly, we also found that 766 circRNAs were differentially expressed (553 up-regulated and 213 down-regulated) in the comparison group of 0 h vs 32 h. The venn analysis was performed to determine the common differentially expressed circRNAs among these 3 different comparison groups. The data showed that the changes of 183 common differentially expressed circRNAs (141 up-regulated and 42 down-regulated ones) occurred throughout the nigericin treatment (Fig. 2a). This implied that these circRNAs described by the hierarchical clustering analysis might participate in the nigericin damage (Fig. 2b). Analyzing these 183 circRNAs, 159 ones were sense-overlapping, 22 ones were derived from exons, and only 2 circRNAs were intergenic (Fig. 3a). Besides, the distribution of these circRNAs on the human chromosomes was depicted in Fig. 3b. Among these 183 circRNAs, the information of the top 20 up- or down- regulated circRNAs including the name of circRNAs, human chromosomes distribution, the molecular types and the parental gene symbols of circRNAs were described in Additional file 3: Table S2, Additional file 4: Table S3, Additional file 5: Table S4 and Additional file 6: Table S5.

### Validation for the sequencing data by qRT-PCR

**Fig. 4:**
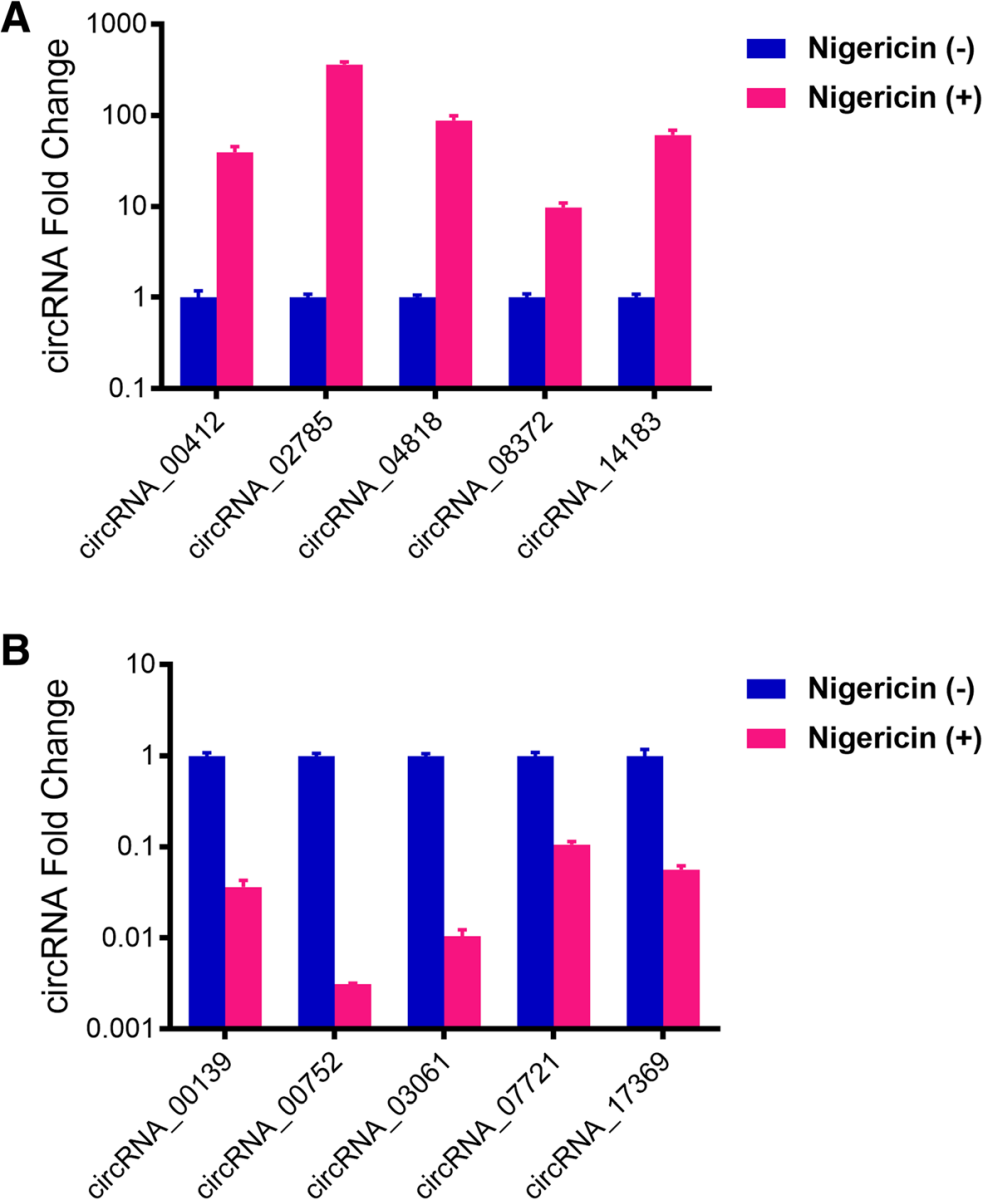
Validation for the sequencing data by qRT-PCR. **a** The expressions of 5 randomly up-regulated circRNAs after 32 h-nigericin exposure were detected by qRT-PCR, compared to the 0 h-nigericin treatment. **b** The expressions of 5 randomly down-regulated circRNAs after 32 h-nigericin exposure were also detected by qRT-PCR. (*P < 0.05)

To verify the reliability of the sequencing data, we randomly selected 10 differentially expressed circRNAs (5 up-regulated and 5 down-regulated ones, and the information of these 10 circRNAs were seen in Additional file 7: Table S6 and Additional file 8: Table S7) and detected their expressions after 32 h-nigericin exposure by qRT-PCR, compared to the 0 h treatment. As expected, the expressing levels of circRNA_00412, circRNA_02785, circRNA_04818, circRNA_08372 and circRNA_14183 were significantly up-regulated at 32 h, compared to those at 0 h (Fig. 4a). On the contrary, the levels of circRNA_00139, circRNA_00752, circRNA_03061, circRNA_07721 and circRNA_17369 were down-regulated (Fig. 4b). These results were consistent well with our sequencing data, which demonstrated the high reliability and validity of the sequencing results.

### The GO and KEGG analysis

**Fig. 5:**
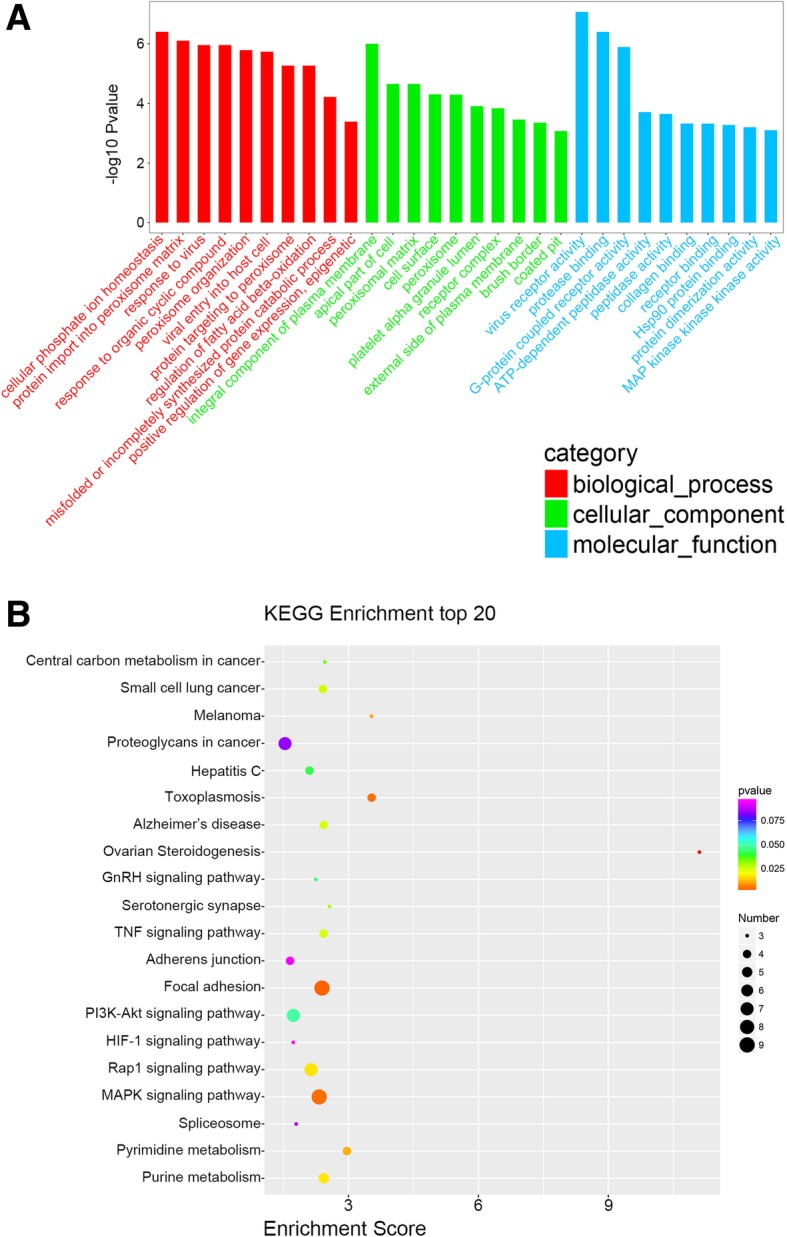
The GO and KEGG analysis were performed by analyzing 164 parental genes of the 183 common differentially expressed circRNAs. **a** A total of 164 parental genes were obtained from the 183 common differentially expressed circRNAs, and the GO analysis presented the top 10 enriched GO terms in the biological process, cellular components and molecular functions. **b** The KEGG pathway analysis exhibited the top 20 most enrichment pathways

In this study, the parental genes of circRNAs were obtained from circBASE database (www.circbase.org/). A total of 164 parental genes were obtained from 183 common differentially expressed circRNAs. The GO analysis revealed that the most significant enriched GO terms in the biological process, cellular components and molecular functions were cellular phosphate ion homeostasis, integral component of plasma membrane and virus receptor activity respectively (Fig. 5a). Meanwhile, the KEGG pathway analysis also exhibited the top 20 most enrichment pathways, including central carbon metabolism in cancer, TNF signaling pathway, Focal adhesion, MAPK signaling pathway, PI3K-Akt signaling pathway, HIF-1 signaling pathway, Pyrimidine metabolism, Purine metabolism and so on (Fig. 5b), which indicated that these pathways might be involved in the nigericin-mediated anti-cancer effects in PC cells. Moreover, to explore the upstream and downstream relationships among these enrichment pathways, a pathway-pathway network including 44 signals was also conducted and described in Additional file 9: Figure S2.

### CircRNA-miRNA interaction network

Accumulating evidence suggest that miRNAs play important roles in various physiological and pathogenesis of cancer as miRNA sponges. To determine the potential functions of these common differentially expressed circRNAs, the interactions between circRNAs and their target miRNAs were theoretically predicted by conserved seed-matching sequence. Our data showed that a total of 88 common differentially expressed circRNAs were found to be combined with 119 binding miRNAs, and the circRNA-miRNA interaction network was presented in Additional file 10: Figure S3. To a single circRNA, it might have more than one binding sites to different miRNAs through the target prediction tool. For example, the up-regulated circRNA_06429 had the potential biding sites of miR-1307-3p, miR-5002-3p, miR-6727-5p and miR-744-5p simultaneously. Similarly, down-regulated circRNA_17075 might sponge miR-3131, miR-326, miR-330-5p and miR-3667-3p as predicted in Additional file 11: Figure S4A. The annotation in Additional file 11: Figure S4B included miRNA response element (MRE) sequences, miRNA seed types and the positions of MREs in the linearized sequences of circRNAs. Besides, a circRNA-miRNA-mRNA network was conducted by our sequencing data. A total of 14 common differentially expressed circRNAs including 9 up-regulated and 5 down-regulated ones were selected (Additional file 12: Figure S5A-B). For instance, circRNA_00858, circRNA_07545, circRNA_16099 and circRNA_17313 might act as sponges of miR-762, which could target IRF7, BAI2, LARS2, CHMP1A, PHLDA1, NACC2 and WWC3. Similarly, circRNA_13271 and circRNA_13579 might be ceRNAs of miR-4459 to regulate the NF2, HIF3A, CCL22, SPC24, PDX1, ORAOV1, SMAGP, DAND5, RAB3A and KRT16 expressions. These target mRNAs might help us better explain the mechanism of nigericin though a potential ceRNA mechanism in PC cells.

## Discussion

PC remains one of the deadliest cancers, with an estimated 53,670 new cases and 43,090 anticipated deaths in the United States 2017 [30]. Despite recent improvements in diagnostic techniques, the prognosis of patients with PC is poor with a 5-year survival rate. The only potential curative treatment is surgical resection, but only 15–20% of patients are eligible for surgery [31]. For patients with advanced and metastatic disease, the median survival remains between 6 and 11 months, and the introduction of combination chemotherapy several decades ago has little impact on these PC patient survival rates. Patients who initially respond to standard of care will eventually relapse with recurrent disease presenting with chemo-resistant carcinomatosis. It is therefore important and urgently need to identify new therapeutic agents that can effectively target PC cells. Researches indicate that nigericin can inhibit various types of cancer cells such as prostate cancer [32], nasopharyngeal carcinoma [10], lung cancer [33] and epithelial ovarian cancer [34], and the underlying biochemical mechanisms for its anti-cancer effects have been comprehensive studied.

Our study is aimed to further elucidate the potential mechanism of nigericin on tumor cells through the high-throughput sequencing. In this study, PC cells (PANC-1 and SW1990) were treated with nigericin at different concentrations for different time periods, and we found that nigericin could inhibit the cell viability in a concentration-dependent and time-dependent manner. Furthermore, we exposed the PC cells to a certain concentrations of nigericin at different time periods, and the expression profile of circRNAs was explored through RNA sequencing. By the venn analysis, 183 common differentially expressed circRNAs were dys-regulated throughout the nigericin damage. A growing number of studies have demonstrated that the aberrant expressions of circRNAs contribute to cell proliferation, metastasis and drug resistance in cancer, and circRNAs are novel RNA molecules with different biological functions and pathological implications. Among these multiple functions, regulating parental gene transcriptions or translations represents one of the most conspicuous functions. For instance, exonic circRNAs might act as “mRNA traps” by shielding the translation start site, leaving a non-coding linear transcript and thereby reducing protein expression [35]. In 2018, Yang et al. showed that circ-FBXW7 and its translated protein FBXW7-185aa have a similar role in glioma carcinogenesis as well as in patient clinical prognosis, which suggested endogenous circRNA could encode a functional protein in human cells [36]. Therefore, in many studies, the GO and KEGG analysis according to these parental genes was performed to further predict the potential functions of dys-regulated circRNAs [37, 38]. In this study, the GO analysis revealed that the most significant enriched GO terms in the biological process, cellular components and molecular functions were cellular phosphate ion homeostasis, integral component of plasma membrane and virus receptor activity respectively. Other top 10 enriched GO terms were also presented, which indicated that these biological processes or molecular functions might be of great significance during the nigericin damage. Moreover, the KEGG pathway analysis provided a deep insight of the mechanism of nigericin in PC cells. The results showed that the potential signaling pathways such as Central carbon metabolism in cancer, Proteoglycans in cancer, GnRH signaling pathway, TNF signaling pathway, Focal adhesion, MAPK signaling pathway, PI3K-Akt signaling pathway, HIF-1 signaling pathway, Pyrimidine metabolism and Purine metabolism might be involved. For instance, among these involved signals, Silvers et al. reported the metabolic profiles of MiaPaCa2 PC cells and indentified the effects of β-lap treatment on central carbon metabolism during PC progression [39]. MAPK and PI3K-Akt signaling pathway had been implicated and frequently activated in the malignant progression of PC, and targeting these two pathways were considered as a strategy for the treatment of PC [40, 41]. In view of the KEGG analysis, we speculated that these related pathways might play an essential role in the nigericin damage. Besides, a pathway-pathway network was also constructed to exhibit the potential regulatory relationships among these pathways, which provided new clues to explore the mechanism of nigericin in tumor cells.

Recent evidence has shown that acting as miRNA sponges to modulate post-transcriptional regulation is another main mechanism of circRNAs in various types of cancers, including prostate cancer [42], oral squamous cell carcinoma [43], gastric cancer [44] and osteosarcoma [45]. Similarly, circRNAs have also raised great interest due to their “microRNA sponges” in PC progression. Qu et al. found that circRHOT1 could bind miR-26b, miR-125a, miR-330 and miR-382 to regulate multiple tumor-associated pathways in PC [46]. A study from Hao et al. showed that circ_0007534 was regarded as an independent prognostic factor for PC patients, and the oncogenic functions of circ_0007534 was partly dependent on its regulation of miR-625 and miR-892b [47]. CircZMYM2 had a similar sponge effect on miR-335-5p and modulated the downstream oncogene JMJD2C in PC [48]. Thus, an assessment of the function of circRNAs as a miRNA sponge to modulate gene expression during nigericin exposure might partly reveal the possible mechanism of this drug in cancers. The interactions between circRNAs and their target miRNAs were theoretically predicted by conserved seed-matching sequence in our study, and a total of 88 common differentially expressed circRNAs were found to be combined with 119 binding miRNAs. To a single circRNA, it might have more than one binding sites to different miRNAs through the target prediction tool. As demonstrated in Additional file 11: Figure S4, the up-regulated circRNA_06429 had the potential biding sites of miR-1307-3p, miR-5002-3p, miR-6727-5p and miR-744-5p simultaneously, and down-regulated circRNA_17075 might sponge miR-3131, miR-326, miR-330-5p and miR-3667-3p. Among these involved miRNAs, for example, miR-1307-3p was an oncogenic miRNA that significantly contributed to breast cancer development and progression [49]. MiR-6727-5p promoted the proliferation, invasion and migration of cervical cancer cells, and inhibited the apoptosis [50]. In contrast, miR-326 was down-regulated in PC patients, and high miR-326 expression prolonged survival likely via the decreasing invasive potential of PC cells [51]. These involved miRNAs were associated with cancer pathogenesis, apoptosis and cell growth, thereby functioning as either tumor suppressors or oncogenes in nigericin damage. Moreover, a circRNA-miRNA-mRNA network was conducted by our sequencing data, in which 14 circRNAs were involved. CircRNA_00858, circRNA_07545, circRNA_16099 and circRNA_17313 might act as sponges of miR-762, which could target IRF7, BAI2, LARS2, CHMP1A, PHLDA1, NACC2 and WWC3. Similarly, circRNA_13271 and circRNA_13579 might be ceRNAs of miR-4459 to regulate the NF2, HIF3A, CCL22, SPC24, PDX1, ORAOV1, SMAGP, DAND5, RAB3A and KRT16 expressions. Though lack of sufficient experimental data in vitro and in vivo and only predicted results were available by bioinformatics analyses, these predicted and target mRNAs might help us better explain the mechanism of nigericin though a potential ceRNA mechanism in PC cells.

In summary, our study firstly performed the high-throughput sequencing to explore the circRNA expression profiles after nigericin exposure at different time points. 183 common differentially expressed circRNAs were identified, and the reliability and validity of the sequencing data was verified by the PCR analysis. The GO analysis according to the parental genes of circRNAs was performed to predict the most significant enriched GO terms in the biological process, cellular components and molecular functions. The KEGG analysis and pathway-pathway network exhibited the signal pathways and their potential regulatory relationships, which provided a deep insight of signals after nigericin exposure. Meanwhile, a potential ceRNA mechanism through a circRNA-miRNA-mRNA network was applied to annotate potential functions of these common differentially expressed circRNAs. To the best of our knowledge, there are no studies discussing the existing or potential mechanism of nigericin by the bioinformatics method. Our data will provide new insight into the molecular mechanism of nigericin toward cancer cells, and facilitate the understanding of nigericin in PC.

## Additional files

Additional file 1: **Table S1.** Primers used for circRNAs analysis. (DOC 38 kb)

Additional file 2: **Figure S1.** The differentially expressed circRNA profile by high-throughput sequencing at different time points. (**a**) The hierarchical clustering analysis in the form of heat map exhibited the changes of circRNAs. (**b**) The scatter plots were used to investigate the circRNA expression profiles. (TIF 5267 kb)

Additional file 3: **Table S2.** The top 20 up-regulated circRNAs ranked by fold changes in our sequencing data. (DOC 85 kb)

Additional file 4: **Table S3.** The top 20 down-regulated circRNAs ranked by fold changes in our sequencing data. (DOC 78 kb)

Additional file 5: **Table S4.** The common up-regulated circRNAs in our sequencing data. (DOC 190 kb)

Additional file 6: **Table S5.** The common down-regulated circRNAs in our sequencing data (DOC 125 kb)

Additional file 7: **Table S6.** Fold changes of 10 validated circRNAs in our sequencing data. (DOC 78 kb)

Additional file 8: **Table S7.** RPM values of the 10 validated circRNAs in our sequencing data. (DOC 89 kb)

Additional file 9: **Figure S2.** A pathway-pathway network described the potential upstream and downstream relationships among 44 enrichment pathways from the KEGG analysis. (TIF 3490 kb)

Additional file 10: **Figure S3.** The putative interactions between miRNAs and circRNAs were evaluated through miRanda, and a circRNA-miRNA interaction network including 88 common differentially expressed circRNAs and 119 binding miRNAs was contructed. The green circles represented the common differentially expressed circRNAs, and the red ones represented the binding miRNAs. (TIF 9333 kb)

Additional file 11: **Figure S4.** The detailed annotation for circRNA-miRNA interaction. (**a**) The bioinformatics prediction showed the target miRNAs of circRNA_06429 and circRNA_17075. (**b**) The detailed annotations including MRE sequences, miRNA seed types and the positions of MREs were predicted by TargetScan and miRanda. (TIF 11416 kb)

Additional file 12: **Figure S5.** A circRNA-miRNA-mRNA network including 14 common differentially expressed circRNAs was conducted. **(a)** 9 up-regulated circRNAs were introduced in the ceRNA network. **(b)** The ceRNA network of 5 down-regulated circRNAs was also built. (TIF 5326 kb)

## Data Availability

The data that support the findings of this study are available from the repository of NCBI Sequence Read Archive (SRA) with the accession numbers: SRR9107550, SRR9107551, SRR9107552 and SRR9107553 (https://www.ncbi.nlm.nih.gov/Traces/study/?acc=PRJNA543685&go=go).

## Abbreviations

ceRNA: competing endogenous RNA
circRNA: circular RNA
ciRNA: circular intronic RNA
DMEM: Dulbecco’s Modified Eagle Medium
FBS: fetal bovine serum
FC: fold change
FDR: false discovery rate
GO: gene ontology
KEGG: Kyoto Encyclopedia of Genes and Genomes
miRNA: microRNA
MREs: miRNA response elements
PC: pancreatic cancer
pre-mRNA: precursor mRNA
qRT-PCR: Quantitative real-time PCR
