# Supplementary material for: High-throughput sequencing of circRNAs reveals novel insights into mechanisms of nigericin in pancreatic cancer
Source: BMC Genomics. 2019 Sep 18;20:716. doi: 10.1186/s12864-019-6032-3 (PMC6749718; doi:10.1186/s12864-019-6032-3)
Supplement: Supplementary file 1 — Table S1. Primers used for circRNAs analysis. (DOC 38 kb) [file 12864_2019_6032_MOESM1_ESM.doc]

**Supplementary Table 1: Primers used for circRNAs analysis**

| **CircRNA** | **Sense** | **Anti-sense** | **Product size** |
| --- | --- | --- | --- |
| circ_00412 | CTGTCAACTCAGCTGCCCTCT | TGGTCATGTCCCTGGATAACTG | 188 |
| circ_02785 | GAGATGTGCTGCTACTGCTTCG | ATTCCCGTGACTGTGCGTTC | 235 |
| circ_04818 | AGAATCCCACTGAAGAAAATAGGCA | ATCTCCATTTCATCTTCTTGACCCT | 235 |
| circ_08372 | AGAACGGTCAAGAAAGAGTAGAAG | AAATCCACTGAACGCAGAGAA | 158 |
| circ_14183 | GGGAATAAGCCCCCAAAGG | TGATAGTCGTTGCGGATGTCG | 167 |
| circ_00139 | AGAGGCTGGAGTGGTGGAA | GAAAGTCAGCTCCGAAATGG | 181 |
| circ_00752 | ACTTGTTACCATTTGTGGGACTGAA | TTCTTCAAATCCCAAGGAGTGTCA | 235 |
| circ_03061 | AGTGACAGAGAATACCCACGCCT | TTGATTGTTGTTGCGGGTTTCT | 207 |
| circ_07721 | GACTCAAAGGAGGCTATGGAAA | CGAAGATCCCTCTCTGTTGTGTAC | 199 |
| circ_17369 | CGTGGAATGAAATCCGAAAC | CTTGTGAACACTGGGGTCGTA | 260 |
| GAPDH | GGAGCGAGATCCCTCCAAAAT | GGCTGTTGTCATACTTCTCATGG | 197 |
