# Supplementary figures and images for: High-throughput sequencing of circRNAs reveals novel insights into mechanisms of nigericin in pancreatic cancer
Source: BMC Genomics. 2019 Sep 18;20:716. doi: 10.1186/s12864-019-6032-3 (PMC6749718; doi:10.1186/s12864-019-6032-3)

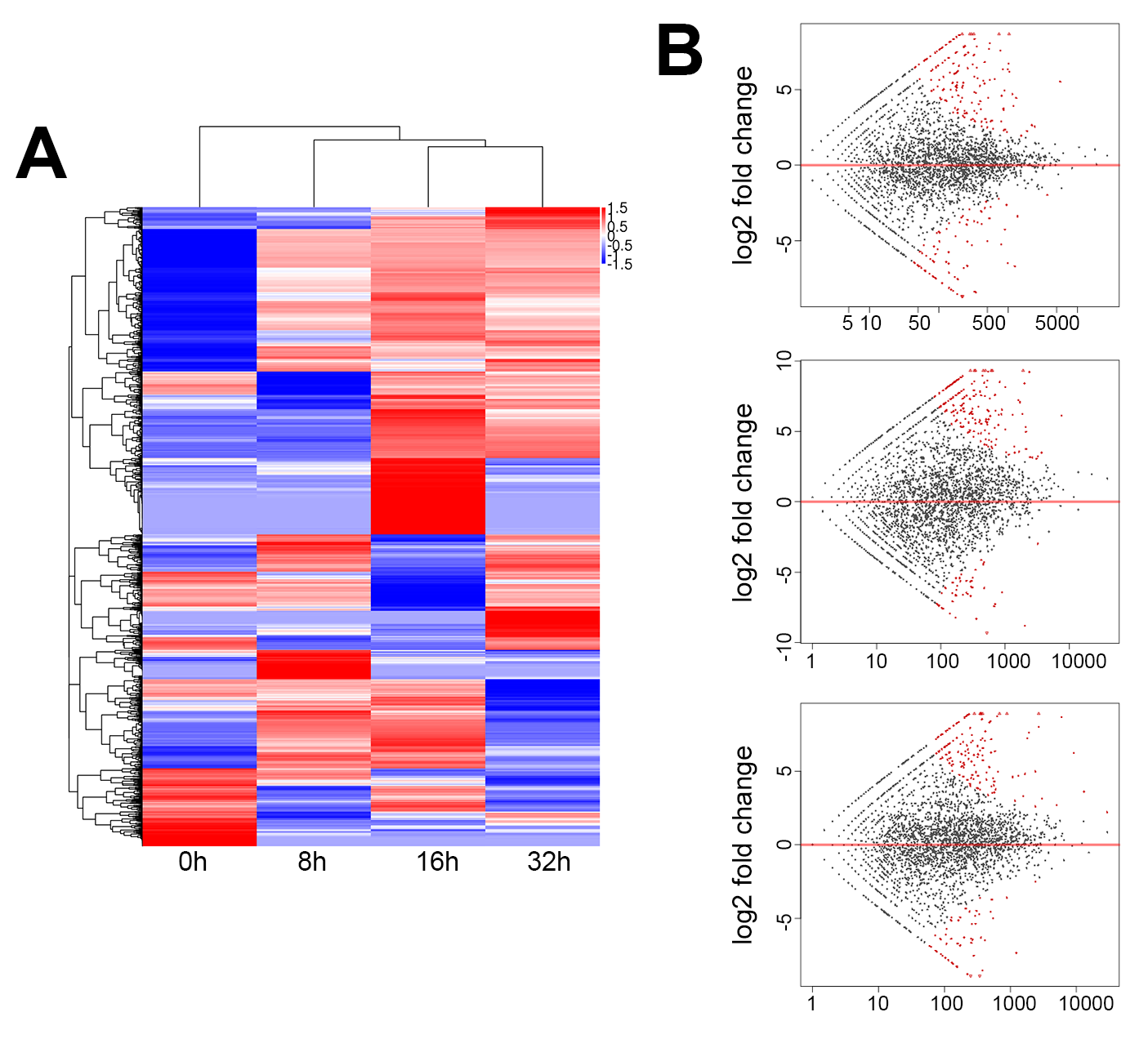

Supplement: Supplementary file 2 — Figure S1. The differentially expressed circRNA profile by high-throughput sequencing at different time points. (a) The hierarchical clustering analysis in the form of heat map exhibited the changes of circRNAs. (b) The scatter plots were used to investigate the circRNA expression profiles. (TIF 5267 kb) [file 12864_2019_6032_MOESM2_ESM.tif]

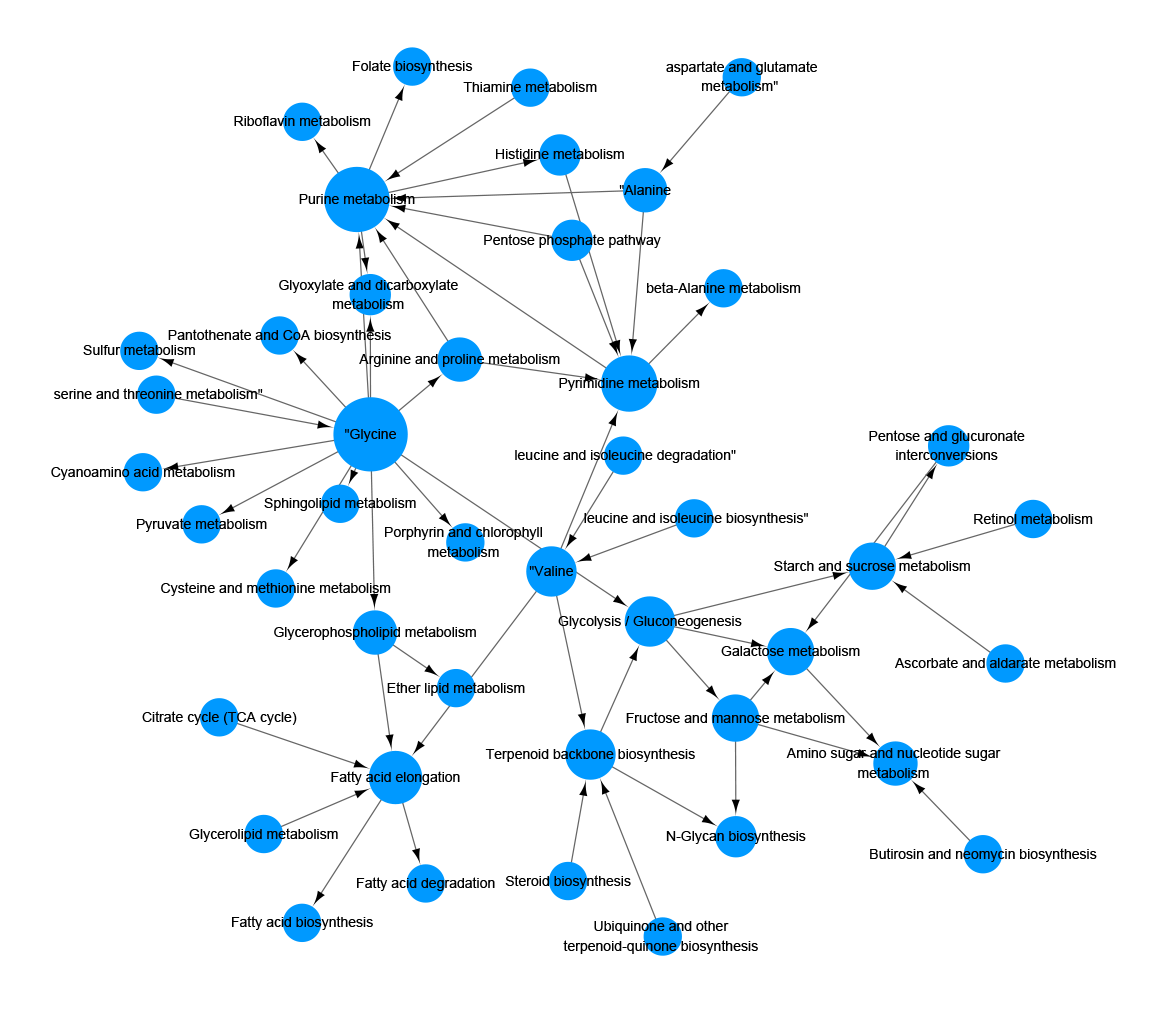

Supplement: Supplementary file 9 — Figure S2. A pathway-pathway network described the potential upstream and downstream relationships among 44 enrichment pathways from the KEGG analysis. (TIF 3490 kb) [file 12864_2019_6032_MOESM9_ESM.tif]

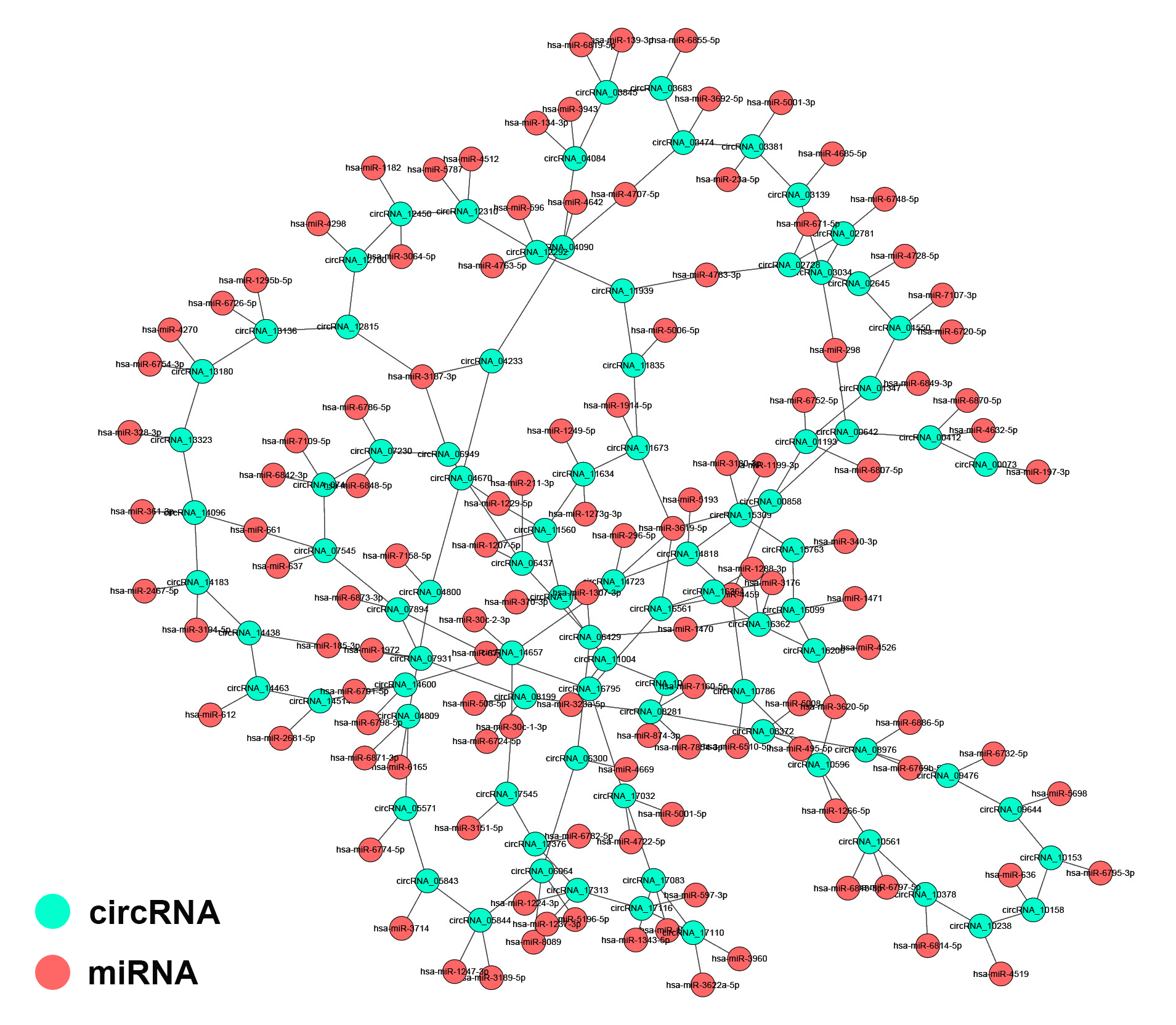

Supplement: Supplementary file 10 — Figure S3. The putative interactions between miRNAs and circRNAs were evaluated through miRanda, and a circRNA-miRNA interaction network including 88 common differentially expressed circRNAs and 119 binding miRNAs was contructed. The green circles represented the common differentially expressed circRNAs, and the red ones represented the binding miRNAs. (TIF 9333 kb) [file 12864_2019_6032_MOESM10_ESM.tif]

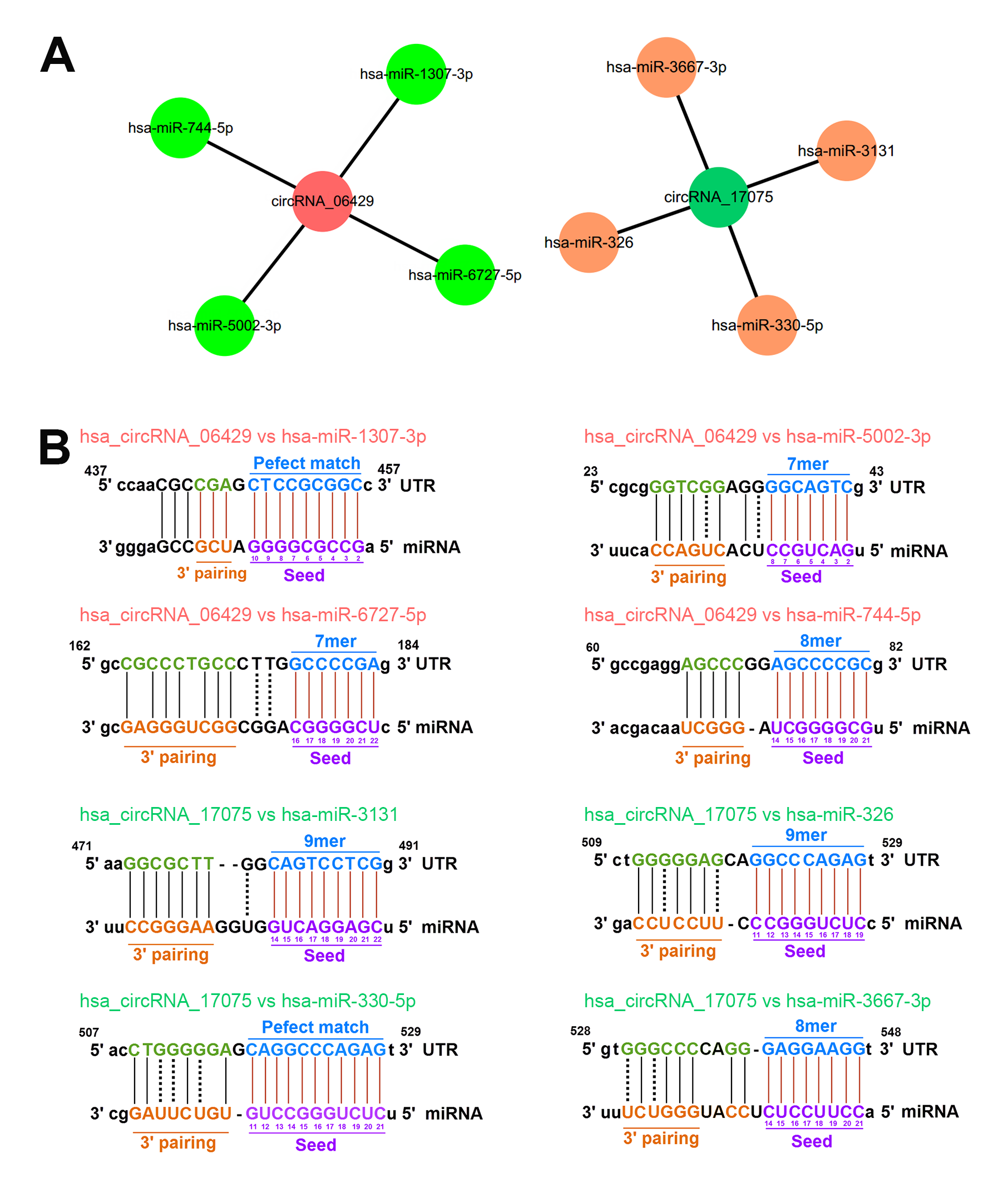

Supplement: Supplementary file 11 — Figure S4. The detailed annotation for circRNA-miRNA interaction. (a) The bioinformatics prediction showed the target miRNAs of circRNA_06429 and circRNA_17075. (b) The detailed annotations including MRE sequences, miRNA seed types and the positions of MREs were predicted by TargetScan and miRanda. (TIF 11416 kb) [file 12864_2019_6032_MOESM11_ESM.tif]

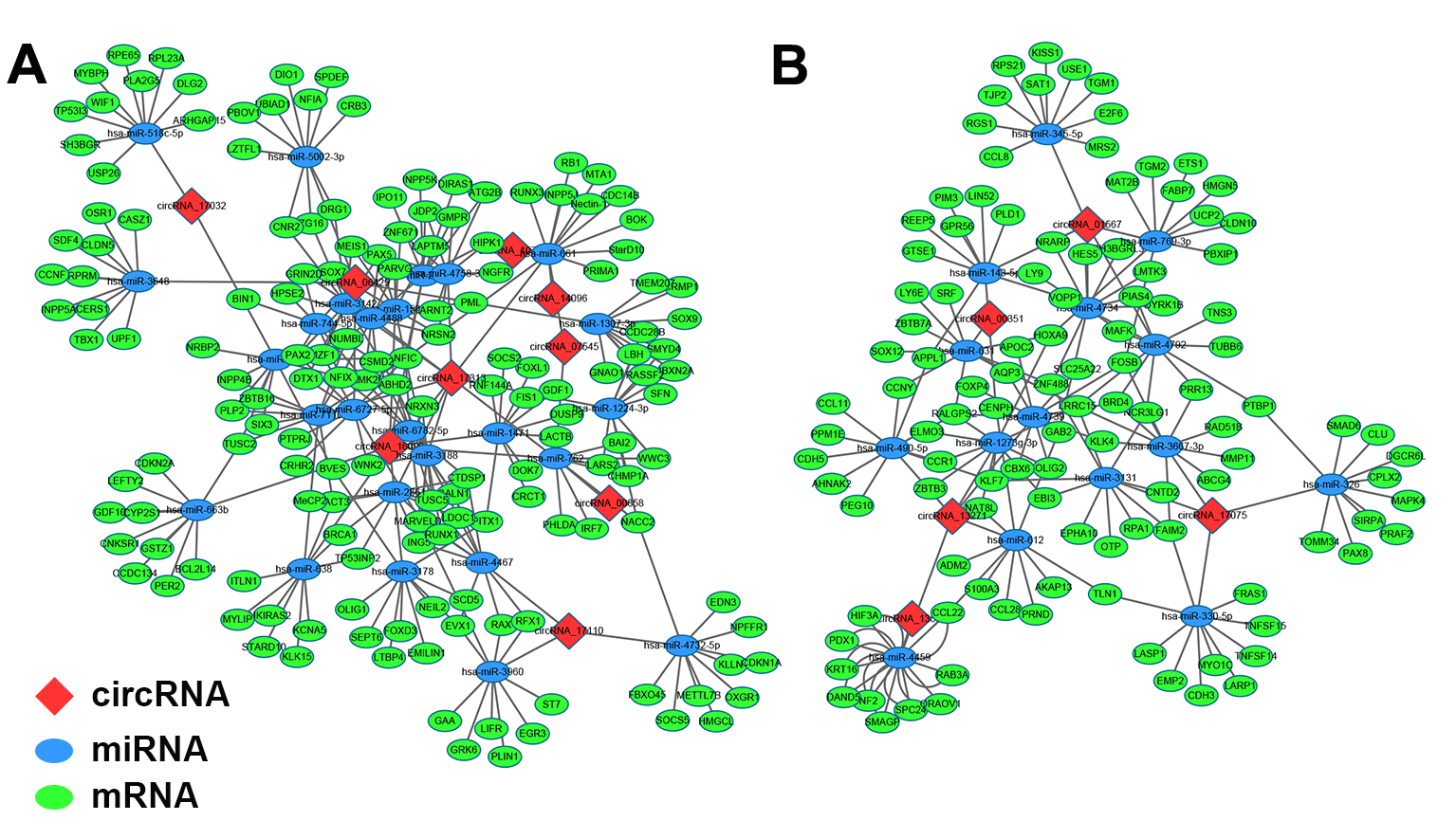

Supplement: Supplementary file 12 — Figure S5. A circRNA-miRNA-mRNA network including 14 common differentially expressed circRNAs was conducted. (a) 9 up-regulated circRNAs were introduced in the ceRNA network. (b) The ceRNA network of 5 down-regulated circRNAs was also built. (TIF 5326 kb) [file 12864_2019_6032_MOESM12_ESM.tif]
