# Supplementary material for: High-throughput sequencing of circRNAs reveals novel insights into mechanisms of nigericin in pancreatic cancer
Source: BMC Genomics. 2019 Sep 18;20:716. doi: 10.1186/s12864-019-6032-3 (PMC6749718; doi:10.1186/s12864-019-6032-3)
Supplement: Supplementary file 3 — Table S2. The top 20 up-regulated circRNAs ranked by fold changes in our sequencing data. (DOC 85 kb) [file 12864_2019_6032_MOESM3_ESM.doc]

**Supplementary Table 2:** The top 20 up-regulated circRNAs ranked by fold changes in our sequencing data

| **CircRNA Chrom Type Gene symbol foldChange** |
| --- |
| circRNA_00073 NC_000001.11 sense-overlapping RPL22 Inf  circRNA_17683 NC_000023.11 sense-overlapping RPS4X Inf  circRNA_08372 NC_000007.14 sense-overlapping DNAJB6 Inf  circRNA_06429 NC_000005.10 exonic DUSP1 Inf  circRNA_17376 NC_000022.11 sense-overlapping LIF Inf  circRNA_04818 NC_000003.12 sense-overlapping ATP13A3 Inf  circRNA_09222 NC_000009.12 exonic ZCCHC7 Inf  circRNA_03683 NC_000003.12 sense-overlapping SLC4A7 Inf  circRNA_04090 NC_000003.12 sense-overlapping FLNB Inf  circRNA_13108 NC_000014.9 sense-overlapping EIF2S1 Inf  circRNA_00858 NC_000001.11 sense-overlapping FUBP1 Inf  circRNA_11004 NC_000011.10 sense-overlapping CELF1 Inf  circRNA_13847 NC_000015.10 sense-overlapping HACD3 Inf  circRNA_00642 NC_000001.11 sense-overlapping FAF1 Inf  circRNA_03845 NC_000003.12 sense-overlapping CDCP1 Inf  circRNA_01704 NC_000002.12 sense-overlapping ADAM17 Inf  circRNA_09476 NC_000009.12 sense-overlapping PTBP3 Inf  circRNA_04680 NC_000003.12 sense-overlapping ACTL6A Inf  circRNA_07010 NC_000006.12 sense-overlapping ASCC3 Inf  circRNA_07545 NC_000007.14 sense-overlappin SNX8 Inf |

Inf: Infinite
