# Supplementary material for: High-throughput sequencing of circRNAs reveals novel insights into mechanisms of nigericin in pancreatic cancer
Source: BMC Genomics. 2019 Sep 18;20:716. doi: 10.1186/s12864-019-6032-3 (PMC6749718; doi:10.1186/s12864-019-6032-3)
Supplement: Supplementary file 4 — Table S3. The top 20 down-regulated circRNAs ranked by fold changes in our sequencing data. (DOC 78 kb) [file 12864_2019_6032_MOESM4_ESM.doc]

**Supplementary Table 3:** The top 20 down-regulated circRNAs ranked by fold changes in our sequencing data

| **CircRNA Chrom Type Gene symbol foldChange** |
| --- |
| circRNA_12123 NC_000012.12 sense-overlapping TMPO 0  circRNA_04527 NC_000003.12 sense-overlapping GMPS 0  circRNA_05657 NC_000005.10 sense-overlapping NUP155 0  circRNA_04526 NC_000003.12 sense-overlapping GMPS 0  circRNA_04423 NC_000003.12 sense-overlapping ATR 0  circRNA_06974 NC_000006.12 sense-overlapping CASP8AP2 0  circRNA_06178 NC_000005.10 sense-overlapping DDX46 0  circRNA_01567 NC_000001.11 sense-overlapping SNAP47 0  circRNA_10666 NC_000010.11 sense-overlapping MKI67 0  circRNA_08832 NC_000008.11 sense-overlapping TAF2 0  circRNA_00351 NC_000001.11 sense-overlapping PHACTR4 0  circRNA_02071 NC_000002.12 sense-overlapping MTA3 0  circRNA_00752 NC_000001.11 sense-overlapping USP1 0  circRNA_00533 NC_000001.11 sense-overlapping CTPS1 0  circRNA_06469 NC_000005.10 exonic HNRNPAB 0  circRNA_14838 NC_000017.11 sense-overlapping SRR 0  circRNA_09391 NC_000009.12 sense-overlapping XPA 0  circRNA_17369 NC_000022.11 sense-overlapping NF2 0  circRNA_08833 NC_000008.11 sense-overlapping TAF2 0  circRNA_10290 NC_000010.11 sense-overlapping BTAF1 0 |
