# Supplementary material for: High-throughput sequencing of circRNAs reveals novel insights into mechanisms of nigericin in pancreatic cancer
Source: BMC Genomics. 2019 Sep 18;20:716. doi: 10.1186/s12864-019-6032-3 (PMC6749718; doi:10.1186/s12864-019-6032-3)
Supplement: Supplementary file 5 — Table S4. The common up-regulated circRNAs in our sequencing data. (DOC 190 kb) [file 12864_2019_6032_MOESM5_ESM.doc]

**Supplementary Table 4:** The common up-regulated circRNAs in our sequencing data

| **CircRNA baseMean_0h baseMean_8h baseMean_16h baseMean_32h baseMean foldChange** |
| --- |
| circRNA_00073 0 4922 4897 8435 6085 Inf  circRNA_00403 0 131 273 159 188 Inf  circRNA_00412 146 2233 4107 4642 3661 25.1  circRNA_00528 4 1080 449 833 788 196.9  circRNA_00642 0 591 1190 949 910 Inf  circRNA_00858 0 995 1109 888 997 Inf  circRNA_01171 0 464 667 213 448 Inf  circRNA_01193 0 206 464 300 323 Inf  circRNA_01346 0 198 254 198 217 Inf  circRNA_01347 0 248 240 142 210 Inf  circRNA_01348 0 96 189 201 162 Inf  circRNA_01450 5 329 358 450 379 75.8  circRNA_01550 0 81 742 470 431 Inf  circRNA_01704 0 647 718 1034 799 Inf  circRNA_02103 0 191 311 730 411 Inf  circRNA_02138 0 99 177 112 129 Inf  circRNA_02207 605 3795 4782 5392 4656 7.7  circRNA_02429 288 1422 3029 2836 2429 8.4  circRNA_02644 0 524 614 117 418 Inf  circRNA_02645 19 417 995 584 665 35.0  circRNA_02728 55 1702 3709 1088 2166 39.4  circRNA_02777 0 367 385 283 342 Inf  circRNA_02781 0 716 767 206 563 Inf  circRNA_02785 0 377 201 433 337 Inf  circRNA_03034 2 359 199 161 240 119.9  circRNA_03139 0 200 318 215 244 Inf  circRNA_03381 0 286 542 267 365 Inf  circRNA_03474 5 185 452 414 350 70.0  circRNA_03683 0 595 1211 1507 1104 Inf  circRNA_03845 0 183 1985 286 818 Inf  circRNA_04084 0 469 897 100 489 Inf  circRNA_04090 0 967 2106 122 1065 Inf  circRNA_04233 0 228 1086 164 493 Inf  circRNA_04456 239 11049 14771 18058 14626 61.2  circRNA_04469 0 360 189 336 295 Inf  circRNA_04670 0 207 217 172 199 Inf  circRNA_04680 0 475 1518 245 746 Inf  circRNA_04800 0 477 201 114 264 Inf  circRNA_04809 21 1060 1762 5423 2748 130.9  circRNA_04818 0 147 1375 2170 1231 Inf  circRNA_05406 0 189 387 560 379 Inf  circRNA_05571 5 333 1642 759 912 182.3  circRNA_05704 4 384 290 220 298 74.5  circRNA_05761 0 185 197 113 165 Inf  circRNA_05762 0 236 534 517 429 Inf  circRNA_05843 0 430 534 184 383 Inf  circRNA_05844 0 211 331 115 219 Inf  circRNA_06064 0 100 1201 721 674 Inf  circRNA_06122 1 218 243 569 343 343.2  circRNA_06123 1 206 157 161 175 174.6  circRNA_06230 24 1251 1559 3614 2141 89.2  circRNA_06300 0 86 911 192 396 Inf  circRNA_06398 0 152 591 97 280 Inf  circRNA_06429 0 319 915 3841 1692 Inf  circRNA_06437 0 559 977 562 699 Inf  circRNA_06949 0 575 311 624 503 Inf  circRNA_07010 0 531 849 840 740 Inf  circRNA_07230 8 697 602 553 617 77.1  circRNA_07442 4 623 1016 234 625 156.1  circRNA_07443 0 230 461 1008 566 Inf  circRNA_07471 1 392 352 458 401 400.6  circRNA_07499 0 64 432 89 195 Inf  circRNA_07545 0 702 846 660 736 Inf  circRNA_07685 0 226 479 283 329 Inf  circRNA_07894 0 105 453 170 243 Inf  circRNA_07931 0 101 353 151 202 Inf  circRNA_08199 45 981 793 1040 938 20.8  circRNA_08281 9 341 472 392 401 44.6  circRNA_08335 0 103 295 144 181 Inf  circRNA_08372 0 1763 4363 2176 2767 Inf  circRNA_08857 0 252 546 300 366 Inf  circRNA_08976 0 88 188 296 191 Inf  circRNA_09222 0 369 1923 1219 1170 Inf  circRNA_09476 0 583 508 1294 795 Inf  circRNA_09644 0 156 207 210 191 Inf  circRNA_10142 3 704 433 1267 801 267.1  circRNA_10153 2 278 884 238 467 233.5  circRNA_10158 0 1423 267 134 608 Inf  circRNA_10238 0 122 424 109 218 Inf  circRNA_10378 19 2021 1161 3796 2326 122.4  circRNA_10500 0 366 371 190 309 Inf  circRNA_10561 0 114 404 680 399 Inf  circRNA_10578 0 142 521 236 300 Inf  circRNA_10596 0 166 241 127 178 Inf  circRNA_10786 1 637 1219 309 722 722  circRNA_10833 28 2037 3392 841 2090 74.6  circRNA_11004 0 358 1826 727 970 Inf  circRNA_11293 0 154 211 236 201 Inf  circRNA_11560 0 65 636 107 269 Inf  circRNA_11634 0 194 350 182 242 Inf  circRNA_11673 1 1472 3813 1369 2218 2218  circRNA_11791 0 190 310 310 270 Inf  circRNA_11835 1 186 915 759 620 620  circRNA_11939 13 711 3987 1007 1901 146.3  circRNA_12292 0 288 239 296 275 Inf  circRNA_12310 0 347 949 550 615 Inf  circRNA_12450 21 891 1087 491 823 39.2  circRNA_12700 0 73 158 290 174 Inf  circRNA_12815 0 301 538 659 500 Inf  circRNA_13108 0 938 1209 904 1017 Inf  circRNA_13136 0 174 206 153 178 Inf  circRNA_13180 0 156 115 274 182 Inf  circRNA_13238 0 404 400 246 350 Inf  circRNA_13323 0 414 457 689 520 Inf  circRNA_13847 0 504 1203 1049 919 Inf  circRNA_14096 0 132 396 383 303 Inf  circRNA_14183 0 182 351 215 249 Inf  circRNA_14316 8 904 1154 760 939 117.4  circRNA_14438 1 219 329 723 424 424  circRNA_14463 0 236 234 91 187 Inf  circRNA_14512 0 328 257 185 257 Inf  circRNA_14514 0 243 557 155 318 Inf  circRNA_14515 4 385 427 243 352 88  circRNA_14600 0 198 774 538 503 Inf  circRNA_14657 0 108 182 99 130 Inf  circRNA_14723 2 457 667 149 288 144  circRNA_14818 0 842 667 465 658 Inf  circRNA_15309 0 97 301 162 187 Inf  circRNA_15763 0 358 775 330 487 Inf  circRNA_15869 0 263 292 162 239 Inf  circRNA_16041 0 500 340 1216 685 Inf  circRNA_16099 6 515 972 631 706 117.6  circRNA_16206 24 1934 3223 936 2031 84.6  circRNA_16277 0 292 394 621 436 Inf  circRNA_16280 0 703 500 160 454 Inf  circRNA_16362 0 98 193 214 169 Inf  circRNA_16364 1 129 378 210 239 239  circRNA_16561 0 397 1012 325 578 Inf  circRNA_16795 3 484 948 213 548 182.8  circRNA_16974 9 198 397 352 315 35.1  circRNA_17032 0 203 473 356 344 Inf  circRNA_17083 1 121 281 159 187 187  circRNA_17110 40 753 2605 1269 1542 38.6  circRNA_17116 114 2496 4069 3236 3267 28.7  circRNA_17140 0 118 527 220 288 Inf  circRNA_17313 2 179 309 368 285 142.5  circRNA_17322 0 74 186 116 125 Inf  circRNA_17376 0 1556 527 1881 1321 Inf  circRNA_17545 0 476 145 289 304 Inf  circRNA_17648 0 152 511 779 481 Inf  circRNA_17683 0 4908 9146 2728 5594 Inf |

Inf: Infinite
