# Supplementary material for: High-throughput sequencing of circRNAs reveals novel insights into mechanisms of nigericin in pancreatic cancer
Source: BMC Genomics. 2019 Sep 18;20:716. doi: 10.1186/s12864-019-6032-3 (PMC6749718; doi:10.1186/s12864-019-6032-3)
Supplement: Supplementary file 6 — Table S5. The common down-regulated circRNAs in our sequencing data (DOC 125 kb) [file 12864_2019_6032_MOESM6_ESM.doc]

**Supplementary Table 5:** The common down-regulated circRNAs in our sequencing data

| **CircRNA baseMean_0h baseMean_8h baseMean_16h baseMean_32h baseMean foldChange** |
| --- |
| circRNA_00122 167 0 0 0 0 0  circRNA_00139 153 0 0 1 0.33 0.002  circRNA_00351 261 0 0 0 0 0  circRNA_00533 223 0 0 0 0 0  circRNA_00752 224 0 0 0 0 0  circRNA_01182 138 0 0 0 0 0  circRNA_01567 309 0 0 0 0 0  circRNA_01714 139 0 0 0 0 0  circRNA_02071 261 0 0 0 0 0  circRNA_02245 972 30 0 41 23.67 0.02  circRNA_03061 257 0 3 0 1 0.004  circRNA_04423 367 0 0 0 0 0  circRNA_04526 379 0 0 0 0 0  circRNA_04527 584 0 0 0 0 0  circRNA_04597 142 0 0 0 0 0  circRNA_04672 549 0 10 0 3.33 0.006  circRNA_05657 415 0 0 0 0 0  circRNA_05810 677 49 0 0 16.33 0.02  circRNA_06178 313 0 0 0 0 0  circRNA_06469 214 0 0 0 0 0  circRNA_06974 323 0 0 0 0 0  circRNA_07232 164 0 0 0 0 0  circRNA_07421 305 0 1 1 0.67 0.002  circRNA_07721 251 0 0 3 1 0.004  circRNA_08355 144 1 0 0 0.33 0.002  circRNA_08465 162 0 0 0 0 0  circRNA_08522 211 0 0 4 1.33 0.006  circRNA_08832 276 0 0 0 0 0  circRNA_08833 177 0 0 0 0 0  circRNA_09391 189 0 0 0 0 0  circRNA_10290 170 0 0 0 0 0  circRNA_10319 748 88 23 2 37.67 0.05  circRNA_10666 307 0 0 0 0 0  circRNA_10994 712 0 0 9 3 0.004  circRNA_11370 164 0 0 0 0 0  circRNA_12123 595 0 0 0 0 0  circRNA_13271 653 2 9 25 12 0.02  circRNA_13579 174 1 0 2 1 0.06  circRNA_14838 199 0 0 0 0 0  circRNA_17075 163 0 0 0 0 0  circRNA_17369 189 0 0 0 0 0  circRNA_17796 797 0 2 0 0.67 0 |
