# Supplementary material for: High-throughput sequencing of circRNAs reveals novel insights into mechanisms of nigericin in pancreatic cancer
Source: BMC Genomics. 2019 Sep 18;20:716. doi: 10.1186/s12864-019-6032-3 (PMC6749718; doi:10.1186/s12864-019-6032-3)
Supplement: Supplementary file 7 — Table S6. Fold changes of 10 validated circRNAs in our sequencing data. (DOC 78 kb) [file 12864_2019_6032_MOESM7_ESM.doc]

**Supplementary Table 6:** Fold changes of 10 validated circRNAs in our sequencing data

| **CircRNA baseMean_0h baseMean_8h baseMean_16h baseMean_32h baseMean foldChange** |
| --- |
| circRNA_00412 146 2233 4107 4642 3661 25.1  circRNA_02785 0 377 201 433 337 Inf  circRNA_04818 0 147 1375 2170 1231 Inf  circRNA_08372 0 1763 4363 2176 2767 Inf  circRNA_14183 0 182 351 215 249 Inf  circRNA_00139 153 0 0 1 0.33 0.002  circRNA_00752 224 0 0 0 0 0  circRNA_03061 257 0 3 0 1 0.004  circRNA_07721 251 0 0 3 1 0.004  circRNA_17369 189 0 0 0 0 0 |

Inf: Infinite
