# Supplementary material for: High-throughput sequencing of circRNAs reveals novel insights into mechanisms of nigericin in pancreatic cancer
Source: BMC Genomics. 2019 Sep 18;20:716. doi: 10.1186/s12864-019-6032-3 (PMC6749718; doi:10.1186/s12864-019-6032-3)
Supplement: Supplementary file 8 — Table S7. RPM values of the 10 validated circRNAs in our sequencing data. (DOC 89 kb) [file 12864_2019_6032_MOESM8_ESM.doc]

**Supplementary Table 7:** RPM values of the 10 validated circRNAs in our sequencing data

| **CircRNA 0h 8h 16h 32h** |
| --- |
| circRNA_00412 16.85062 207.0805 425.8806 451.116  circRNA_02785 7.28E-11 21.62778 12.87691 25.99748  circRNA_04818 3.24E-08 7.131196 74.68055 110.484  circRNA_08372 0.002065283 130.331 360.6019 168.5603  circRNA_14183 0.0001373259 8.509771 18.33738 10.55879  circRNA_00139 12.12835 1.39E-09 4.34E-12 0.0729014  circRNA_00752 15.31075 7.56E-16 5.65E-09 0.005468245  circRNA_03061 16.54608 8.79E-09 0.1977886 0.001001855  circRNA_07721 26.14245 2.02E-09 2.40E-21 0.2683411  circRNA_17369 10.86877 2.29E-07 0.0006556197 6.75E-05 |
